# Standardized, risk-adapted induction therapy in kidney transplantation

**DOI:** 10.1007/s40620-023-01746-1

**Published:** 2023-09-09

**Authors:** Felix Eisinger, Thomas Mühlbacher, Ario Na, Karina Althaus, Silvio Nadalin, Andreas L. Birkenfeld, Nils Heyne, Martina Guthoff

**Affiliations:** 1 Department of Diabetology, Endocrinology, Nephrology, University of Tübingen, Otfried-Müller Str. 10, 72076 Tübingen, Germany; 2 Institute for Diabetes Research and Metabolic Diseases, Helmholtz Center Munich, University of Tübingen, Tübingen, Germany; 3 German Center for Diabetes Research (DZD e.V.), Neuherberg, Germany; 4 Department of General-, Visceral- and Transplant Surgery, University of Tübingen, Tübingen, Germany; 5 Center for Clinical Transfusion Medicine, University of Tübingen, Tübingen, Germany

**Keywords:** Kidney transplantation, Induction therapy, Immunological risk, Standard operating procedure

## Abstract

**Background:**

The choice of induction therapy in kidney transplantation is often non-standardized and centre-specific. Clinicians can choose between depleting and non-depleting antibodies, which differ in their immunosuppressive capacity and the concomitant risk of infection. We herein present a standardized risk-stratified algorithm for induction therapy that might help to balance the risk of rejection and/or serious infection.

**Methods:**

Prior to kidney transplantation, patients were stratified into low-risk, intermediate-risk or high-risk according to our protocol based on immunologic risk factors. Depending on their individual immunologic risk, patients received basiliximab (low risk), antithymocyte globulin (intermediate risk) or low-dose alemtuzumab (high risk) for induction therapy. We analysed the results after 3 years of implementation of our risk-stratified induction therapy protocol at our kidney transplant centre.

**Results:**

Between 01/2017 and 05/2020, 126 patients were stratified in accordance with our protocol (low risk/intermediate risk/high risk: 69 vs. 42 vs. 15 patients). The median follow-up time was 1.9 [1.0–2.5] years. No significant difference was observed in rejection rate and allograft survival (low risk/intermediate risk/high risk: 90.07% vs. 80.81% vs. 100% after 3 years (p > 0.05)) among the groups. The median eGFR at follow-up was (low risk/intermediate risk/high risk) 47 [33–58] vs 58 [46–76] vs 44 [22–55] ml/min/1.73 m^2^. Although the rate of viral and bacterial infections did not differ significantly, we observed a higher rate of opportunistic fungal infections with alemtuzumab induction.

**Conclusions:**

Our strategy offers facilitated and individualized choice of induction therapy in kidney transplantation. We propose further evaluation of our algorithm in prospective trials.

## Introduction

**Fig. 1:**
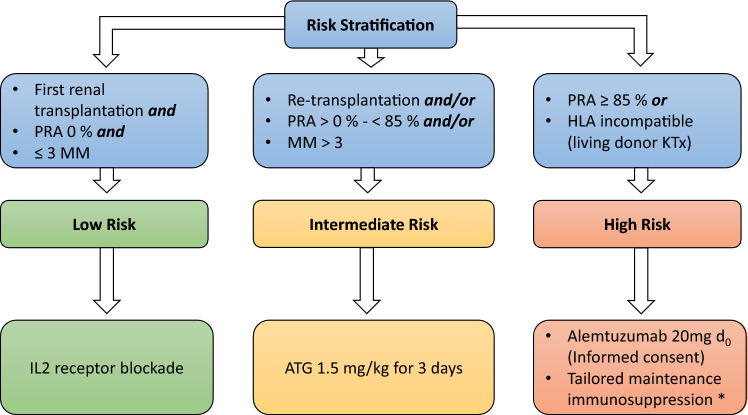
Tübingen Induction Algorithm. Flow chart of immunological risk stratification prior to transplantation and induction therapy algorithm based on immunological risk. The threshold of HLA mismatches defining immunological risk was based on haploidentical HLA matches, taking only HLA-A, -B and -DR antigens into account. *MMF commenced when lymphocyte count > 5% or 200/µl

Transplant guidelines recommend the use of induction therapy in the majority of patients undergoing kidney transplantation [1, 2]. In clinical routine, the choice of induction therapy for the individual patient is challenging and dependent on immunological risk. To facilitate the decision at the time of organ allocation, we developed an innovative, standardized algorithm for risk stratification and subsequent choice of induction therapy (Fig. 1).

The antibodies used in our algorithm can be categorized into lymphocyte-depleting (polyclonal antithymocyte globulin (ATG) and anti-CD52 alemtuzumab) and non-depleting antibodies (basiliximab). Antithymocyte globulin and alemtuzumab lead to complete lymphocyte depletion in peripheral blood and induce stronger immunosuppression than basiliximab. The 2009 KDIGO guideline recommends the use of IL2 receptor antibodies as first-line therapy in low immunologic risk patients [1]. In patients at higher immunologic risk, ATG is more effective than basiliximab in preventing rejection, graft loss and death [3]. However, due to the increase in immunosuppression, the incidence of infections is higher in patients treated with ATG than in those who receive basiliximab [3]. Alemtuzumab leads to a dose-dependent sustained lymphocyte depletion in peripheral blood for several months [4]. We therefore primarily use it for patients with a very high immunological risk. We herein present our algorithm for a risk-adapted induction therapy in kidney transplantation and report first data on clinical outcome.

## Materials and methods

Prior to kidney transplantation, recipients were stratified into immunologic low-risk (LR), intermediate-risk (IR) or high-risk (HR) groups depending on their panel reactive antibodies (PRA), number of HLA-mismatches (based on haploidentical HLA matches, taking Eurotransplant typing only HLA-A, -B and -DR antigens at that time-point into account), presence of preformed donor-specific HLA antibodies (only HLA-incompatible living donor kidney transplantation with prior preconditioning) and previous kidney transplantations. Our algorithm was defined in an interdisciplinary meeting of experts in the field of kidney transplantation, including surgeons, nephrologists and transfusion medicine specialists.

Patients received induction therapy with basiliximab 20 mg on day 0 and day 4 (LR group), ATG 1.5 mg/kg bodyweight for three subsequent days (from day 0 to day 2) (IR group) or low-dose alemtuzumab 20 mg as a single dose on day 0 (HR group). In the LR and IR group, maintenance immunosuppression consisted of low-dose prednisolone, tacrolimus and mycophenolate mofetil (MMF). In the HR group, MMF was withheld until the lymphocyte count reached > 5% of leukocytes or > 200/µl absolute [4]. Standard infection prophylaxes (PJP, CMV) were performed.

We analysed all kidney transplant recipients who had been stratified in accordance with the standardized protocol between 01/2017 and 05/2020 at our centre. Children under the age of 18 were not included. The analysis was conducted in accordance with the Declaration of Helsinki and approved by the local institutional review board (796/2020BO2). Data are given as median [interquartile range]. All statistical analyses were performed using the JMP 16.2.0 statistical software package. A chi-square test was used to analyse relationships between categorical variables. Ordinal and interval-scaled variables were assessed with the Kruskal–Wallis test. Kaplan–Meier curves were compared using the log rank test. A *p* value of less than 5% was set as threshold of significance.

## Results

### Patient and transplant characteristics

In the observation period from 01/2017 to 05/2020, 126 patients were analysed over the median follow-up of 1.9 [1.0–2.5] years (Table 1).

**Table 1:** Patient characteristics and results

	LR	IR	HR	*p*
Total number	69	42	15	
Gender (f/m)	23/46	19/23	8/7	0.238
Age (years)	62 [50–67]	49 [40.3–62]	59 [50.5–65]	0.018
BMI (kg/m^2^)	25.7 [22.6–29.8]	23.2 [20.7–26.5]	24.4 [22.9–28.4]	0.028
Follow-up period (years)	1.7 [1.0–2.6]	1.7 [1.0–2.1]	1.9 [1.2–2.6]	0.711
# of transplantation (*n*)				
1st	64	35	7	< 0.001
2nd	5	7	5	0.021
3rd or more	0	0	3	< 0.001
DD/LD donor transplantation	50/19	27/15	13/2	0.248
Warm ischaemia time (h)	0.5 [0.4–0.6]	0.5 [0.4–0.6]	0.5 [0.4–0.6]	0.880
Cold ischaemia time (h)	6.5 [2.8–11.6]	5.6 [2.8–11.5]	10.8 [8.4–11.7]	0.171
Plasmapheresis/immunoadsorption prior to transplantation	5 (7%)	4 (10%)	6 (40%)	0.0015
PRA max. (%)	0 [0–0]	0 [0–0]	78.9 [55.5–86.2]	< 0.001
MM HLA class I (n)	4 [3–5]	5 [4–5]	3 [3–4]	0.086
MM HLA class II (n)	2 [2–3]	3 [2–4]	2 [1–2]	0.023
Results				
eGFR in ml/min/1.73 m^2^				
At discharge	37 [30.25–52]	47.5 [33–68.75]	41 [16–50]	0.023
At follow-up, all modalities	47 [33–58]	58 [46–76]	44 [22–55]	0.008
Living donor at follow-up	56 [49–64]	59 [52–78]	26 [25–27]	0.026
Deceased donor at follow-up	42.5 [29.5–57]	56 [30–79]	46 [17.5–57]	> 0.05
Delayed graft function	11 (16%)	2 (5%)	4 (27%)	> 0.05
Leukopenia	39 (57%)	32 (76%)	14 (93%)	0.0075
Lymphopenia	60 (87%)	42 (100%)	15 (100%)	< 0.001
Severe lymphopenia	8 (11.6%)	35 (83%)	15 (100%)	< 0.001
Time of recovery from severe lymphopenia in days	N/A	13 [7–23]	114 [83–132]	< 0.001
Infectious complications				
CMV infections	7 (10%)	6 (14%)	1 (7%)	> 0.05
EBV replication	5 (7%)	4 (10%)	0 (0%)	> 0.05
PVAN	5 (7%)	4 (10%)	0 (0%)	> 0.05
PJP pneumonia	2	1	1	> 0.05
Urinary tract infection rates	0.5 [0–1]	0 [0–0.7]	0.7 [0.5–1.3]	> 0.05
Systemic opportunistic fungal	1 (1.4%)	0 (0%)	2 (14%)	0.04
Prediabetes post-Tx (%)	55	67	71	> 0.05
PTDM (%)	18	11	25	> 0.05

Data are given as median [interquartile range]

According to the protocol, 69 patients were stratified to the LR group, 42 to the IR and 15 patients to the HR group. A high proportion of patients in the IR group received living donor kidney transplantation (LR/IR/HR: 27.54% vs. 35.71% vs 13.33%), which was, however, not statistically significant.

### Allograft function, rejection and survival

At discharge and at the end of follow up, estimated glomerular filtration rate (eGFR) differed significantly among groups, with 37 vs 47.5 vs 41 ml/min/1.73 m^2^ (LR/IR/HR, *p* = 0.023). The eGFR at follow-up remained superior in the IR group. In a subgroup analysis by transplant modality (living/deceased donor transplantation), no consistent difference in eGFR among the groups could be found.

There was no significant difference in the number of biopsy-proven acute rejections among the groups. During the follow-up period, 7 cases of biopsy-proven acute rejection occurred in the LR-group (10%), 3 cases in the IR-group (7%) and 1 case in the HR-group (7%). The rejection-free survival rate after 1 year and after 3 years did not differ significantly among the groups.

**Fig. 2:**
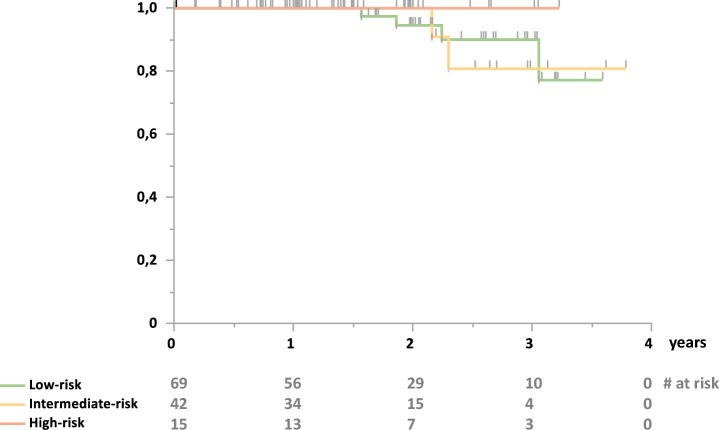
Death-censored allograft survival. Kaplan–Meier estimate of death-censored allograft survival; green: low-risk group, yellow: intermediate-risk group; red: high-risk group

During follow-up, 7 out of 69 allografts (10%) in the LR-group were lost, compared to 2 out of 42 (5%) in the IR-group and none in the HR-group. Allograft survival after 1 year was 100% in every group and, after 3 years, 90.07% vs. 80.81% vs. 100% (LR/IR/HR) (*p* > 0.05) (Fig. 2). Three of the allografts lost in the LR-group were attributed to death with functioning allograft. Causes of death were myocardial infarction and infectious complications (pneumocystis jirovecii pneumonia, cryptococcosis). The other 4 allografts in the LR-group were lost due to infections (2 × bacterial sepsis, 1 × COVID-19, 1 × polyomavirus-associated nephropathy (PVAN)). In the IR-group, both allografts were lost due to PVAN.

### Leukopenia and lymphopenia

In the first year after transplantation, owing to the mechanism of action, patients treated with ATG or alemtuzumab presented with significantly more leukopenia, lymphopenia and severe lymphopenia than patients treated with IL2 receptor antibodies. Time to recovery from severe lymphopenia was significantly longer following alemtuzumab than after ATG (IR/HR: 13 vs 114 days).

### Infectious complications

There was no significant difference in the rate of CMV, EBV and BKV infection among the groups (all *p* > 0.05). Pneumocystis jiroveci pneumonia occurred in 2 patients in the LR-group, in 1 patient in the IR-group and in 1 patient in the HR-group. Urinary tract infection rates did not significantly differ among the groups. The percentage of opportunistic fungal infections was significantly higher in the HR-group than in the other groups (LR/IR/HR: 1.4% vs. 0% vs. 14%, *p* = 0.04).

## Discussion

We herein propose a tailored, risk-stratified induction therapy algorithm for kidney transplantation, enabling a facilitated and individualized choice of induction therapy. Our algorithm is a personal viewpoint, based on longstanding expertise in the field of kidney transplantation. We use universally recognized immunologic risk factors (re-transplantation, HLA typing, PRA levels) to stratify patients according to their immunological risk. Our algorithm provides the great advantage of a highly standardized choice of induction therapy, which reduces the risk of arbitrary decision-making in the transplant setting. In a recent French multicentre analysis, the choice of induction therapy highly varied among the centres and seemed to be based rather on centre-specific practices than on standardized rules [5]. Furthermore, patients were usually stratified into low- or high-risk groups, receiving either basiliximab or ATG. We propose a more refined risk stratification strategy in low-, intermediate- and high-risk patients. Interestingly, in our transplant cohort, there was no relevant difference in biopsy-proven acute rejection rates after one and three years among the three groups, even though there was such a wide range of immunological risk. The overall rejection rates in our cohort (LR: 9.2%, IR: 4.8%, HR: 6.7%) after one year were comparable to those reported in the literature (IL2 induction: 8.4%, T-cell depleting induction: 6.9%) [6].

Surprisingly, allograft function was significantly better, and delayed graft function was numerically lower in the IR-group than in the other groups. There was, however, a non-significant trend towards a higher number of living donor transplantations in the IR-group, which might explain the better outcome of this group. On the whole, allograft survival after one year was similar to allograft survival rates in the literature [7].

**Fig. 3:**
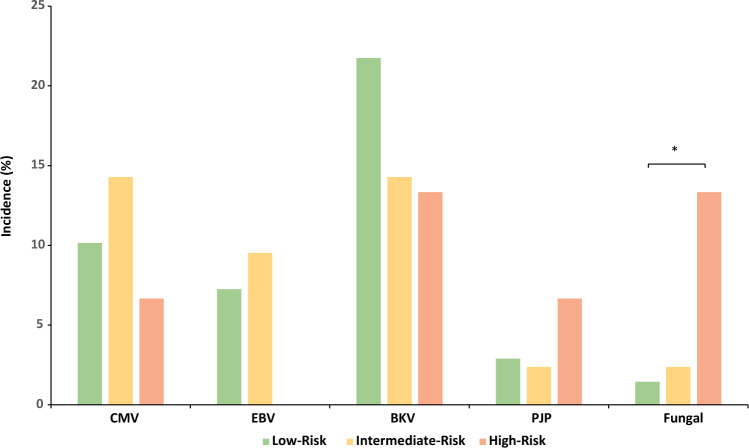
Opportunistic infections. Opportunistic infections according to immunological risk groups; green: low-risk group, yellow: intermediate-risk group; red: high-risk group; *p < 0.05

One central aim of our induction protocol is, besides sufficient rejection prophylaxis, minimization of infectious complications. In a prospective multicentre trial, patients who had received induction therapy with ATG rather than with basiliximab showed a higher rate of infections (85.8% vs. 75.2%) [8]. In a retrospective, five-year analysis comparing alemtuzumab-based induction therapy with ATG- and basiliximab-based protocols, a significantly higher incidence of CMV infection and a trend towards higher rates of bacterial and BKV infection in alemtuzumab-treated patients were found [9]. However, it should be borne in mind that the dosage of alemtuzumab in this study was higher than that used at our centre (30 mg vs 20 mg) [4]. In our study, no significant difference among the various induction treatment groups was observed with regard to CMV and BKV infection rates (Fig. 3). This may be due to our low-dose alemtuzumab scheme. However, our data showed a significantly higher number of systemic opportunistic fungal infections due to *Candida albicans.* It seems plausible that the prolonged severe lymphopenia puts patients on alemtuzumab at higher risk for severe opportunistic infections. Therefore, alemtuzumab as an induction therapy should be limited to high risk patients. We propose emphasizing the possible risks and benefits of the use of alemtuzumab to enable patients to make an informed decision to treatment.

In summary, our algorithm is based on personal expertise in the field of kidney transplantation and our data analysis can only present a low level of evidence as a retrospective single-centre analysis. However, our algorithm is standardized and easily-applicable, based on a readily available risk stratification. We suggest broader implementation of our algorithm and prospective multicentre evaluation to further improve risk-adapted induction therapy in kidney transplantation.

## Data Availability

The data file can be accessed in the Dryad Digital Repository. However, after consultation of the ethics committee, the legal department and the medical faculty, the data file has been restricted in terms of patient demographic and anthropometric variables to ensure anonymity. All measured values relevant to the analysis can be found in the data file. This approach is based on the fact that our cohort is strictly selected from a single university hospital, leading to an otherwise very closely described patient group.
